# Bone Mineral Density and Bone Turnover Marker in a Subclinical Thyrotoxic State in Young Premenopausal Women

**DOI:** 10.7759/cureus.52610

**Published:** 2024-01-20

**Authors:** Chirag LU, Mala Dharmalingam, Manjunath P R, Ganavi Y P, Chitra Selvan, Pramila Kalra

**Affiliations:** 1 Endocrinology and Diabetes, Ramaiah Medical College, Bengaluru, IND; 2 Endocrinology and Diabetes, Bangalore Endocrinology and Diabetes Research Centre, Bengaluru, IND

**Keywords:** exogenous, endogenous, women, premenopausal, dxa, bone turnover markers, subclincal hyperthyroidism

## Abstract

Background: Subclinical thyrotoxicosis (SCH) is characterized by normal serum thyroid hormone levels and low thyrotropin levels. The impact of this condition on the skeletal system may vary depending on its cause, yet the relationship is not fully comprehended in premenopausal women. Studies are scarce about its effects on bone health in our population.

Objectives: This study aims to evaluate the bone mineral density (BMD) and bone turnover markers in premenopausal women with SCH and determine if any differences exist based on the condition's etiology.

Methods: A cross-sectional study was conducted at Ramaiah Medical College involving 36 participants for one year and six months after approval from the Ethics Committee. The carboxy-terminal telopeptide of type I collagen in blood and BMD were measured at the lumbar vertebrae (L1-L4) and femoral neck by dual-energy x-ray absorptiometry (Hologic v 2.0, Hologic, Massachusetts, U.S.). Statistical analysis was done using IBM SPSS Statistics for Windows, Version 20 (Released 2011; IBM Corp., Armonk, New York, United States).

Results: The mean age of the study population was 35.2 ± 7.2 years. The etiology was Graves' disease [n=11 (33.3%)], iatrogenic [n=14(38.8%)], toxic adenoma [n=6 (15.1%)], and multi-nodular goiter [n=5 (15.1%)]. The mean BMI was 23.5 ± 3.8 kg/m^2^, and the mean levels of corrected calcium, phosphorus, and 25 hydroxy-vitamin D were 9.12 ± 0.25 mg/dl, 2.95 ± 0.34 mg/dl, and 29.4 ± 6.4 ng/ml, respectively. The mean BMD at hip and spine was 0.81 ±0.16 g/cm^2^ and 0.92±0.08 g/cm^2^ respectively. The mean Z-score was (-0.02 ± 0.8) and (-0.92± 0.08) at the hip and spine. No significant difference was observed in the BMD at the hip (p = 0.14) or spine (p = 0.44) between the endogenous and exogenous subclinical thyrotoxic subgroups. At the same time, the carboxy-terminal telopeptide of type I collagen was significantly different between the two groups (p<0.05).

Conclusion: In our cross-sectional study of premenopausal women with SCH, BMD at the hip or spine as measured by dual-energy X-ray absorptiometry did not reveal any significant reduction. The subclinical thyrotoxic state may not have an adverse effect on bone health in premenopausal females with sufficient levels of serum 25-hydroxy-vitamin D in the short term.

## Introduction

Subclinical thyrotoxicosis (SCH) state is a condition where serum thyroid hormone levels are within the reference range but serum thyrotropin levels are subnormal [1]. It may be caused by overproduction of endogenous thyroid hormone or excessive ingestion of exogenous thyroid hormone. Endogenous etiology refers to the SCH state caused by Graves disease, toxic multi-nodular goiter, and toxic adenoma.

It is also unclear whether differences in patterns of thyroid hormone levels between endogenous and exogenous subclinical thyrotoxic states result in disparate effects on the skeletal system in premenopausal women [2].

SCH state in the elderly may be associated with bone loss more frequently compared to premenopausal women as age and menopausal status are also significant contributing factors. Endogenous SCH has consistently been correlated with an increased risk of reduced bone mineral density (BMD) in postmenopausal women [3-6].

However, in premenopausal women, multiple studies of premenopausal women with SCH have consistently reported not pathologically decreased BMD levels and its effect on bone health status is not clear [7,8]. Premenopausal women in SCH need a special mention as they are more prone to future complications and detection of early bone loss is vital.

Bone turnover markers (BTMs) are a series of protein or protein derivative biomarkers released during bone remodeling by osteoblasts or osteoclasts. BTMs are a useful adjunct to dual-energy X-ray absorptiometry (DXA) scans for the diagnosis and therapeutic monitoring of bone metabolic disorders. C-terminal telopeptide of type 1 collagen (CTx) is the specific product of cathepsin K-mediated bone resorption, as direct digestion of bone with cathepsin K but not alternative catabolic enzymes, such as matrix metalloproteinases, causes CTx release [9]. It may aid in providing evidence of increased bone resorption in these patients.

There is a lack of data in India regarding these patients and their bone health status. It is essential to study and identify these patients at an early stage for intervention and the development of effective screening protocols for bone health.

## Materials and methods

This cross-sectional study was conducted at the Department of Endocrinology, Ramiah Medical College, over a study period from September 2021 to December 2022. The study aimed to investigate subclinical thyroid states in subjects who met the specified inclusion and exclusion criteria. The Ethics Committee of Ramaiah Medical College approved the study protocol under DRP- IFP 671/2021.

The inclusion criteria for the study included individuals with subclinical hyperthyroidism, encompassing both endogenous and exogenous causes. The age range for eligible participants was between 18 and 50 years, and women who provided consent were enrolled. In addition, subjects were required to have sufficient levels of Vitamin D3 (>20ng/dl).

Exclusion criteria were defined to ensure the study population's homogeneity and to minimize confounding factors. Subjects above the age of 50, pregnant or lactating females, and individuals with a history of chronic kidney disease, diabetes mellitus, chronic steroid therapy, any malignancy, BMI below 17 kg/m^2^, vitamin D3 insufficiency, post-menopausal status, or the use of drugs that lower thyroid-stimulating hormone (TSH) (such as biotin, metformin, dopamine, corticosteroids, or octreotide) were excluded from the study. Moreover, individuals with known neurological and neuromuscular disorders such as stroke, demyelinating disorders, myopathies, or muscular dystrophy were not included in the study population. All female subjects in the study reported regular menstrual cycles with intervals ranging from 22 to 29 days.

After obtaining informed consent from patients who attended the Endocrinology outpatient department, a detailed history and physical examination were done. A blood sample of 10 milliliters was obtained for analysis, including investigations such as serum calcium, phosphorus, albumin, vitamin D3, alkaline phosphatase, thyroid profile, and carboxy-terminal telopeptide of type I collagen (CTx). If vitamin D3 was found insufficient, six weeks of therapy was given with 60,000 IU of vitamin D3, and the patients were reassessed for inclusion criteria.

BMD at the lumbar vertebrae (L1-L4) and femoral neck was assessed using dual-energy x-ray absorptiometry (Hologic v 2.0). BMD was expressed in the units of grams per square centimeter (g/cm^2^). The measurements of vitamin D, TSH, free T4 (fT4), and free T3 (fT3) were made by the electrochemiluminescence immunoassay (ECLIA) method (Roche Cobas 6000 analyzer, Roche Diagnostics, Basel, Switzerland). Bone turnover marker CTx was determined by the chemiluminescence immunoassay (CLIA) method (Roche Cobas E assay CA) on a fasting sample. The assay's minimum and maximum detection level was 10-600 pg/ml.

Sample size

A study conducted by Gürlek et al. in premenopausal women with a subclinical hyperthyroid state has observed that mean femoral neck BMD and lumbar spine BMD was 1.04 (0.8 - 1.29) g/cm^2^ and 1.17 (0.92-1.43) g/cm^2 ^[10]. In the present study expecting a similar result with a 95% confidence interval and 9% relative precision, the study requires a minimum of 27 subjects.

Statistical method

Descriptive statistics of demographical data, femoral BMD, lumbar BMD, and BTMs will be analyzed and summarized in terms of mean with standard deviation. An Independent t-test was used to compare the means of BMD and CTx between endogenous and exogenous subgroups. Spearman’s rank correlation was used to measure the correlation between variables.

## Results

In this study, a total of 36 premenopausal women with SCH were enrolled who satisfied the inclusion criteria. The mean age of the participants was 35.2 years, with a standard deviation of 7.2 years, and the age range of 23 to 46 years.

Regarding the etiology of SCH, it was classified into two main categories: endogenous and exogenous. Among the participants, 22 individuals (61.1%) had endogenous causes, with Graves' disease accounting for 11 cases (33.3%), toxic adenoma for six cases (15.15%), and multi-nodular goiter for five cases (15.1%). On the other hand, 14 individuals (38.8%) had exogenous causes of SCH. Twenty patients (90.1 %) in the endogenous group and eleven patients (78.5%) in the exogenous group had received prior supplementation with vitamin D. The baseline characteristics of the patients are shown in Table 1. The mean BMD and CTx in both groups are shown in Table 2.

**Table 1:** Baseline characteristics of the study group The data has been represented as Mean±SD, etc. The p-value is considered significant at <0.05.

Parameter	Endogenous subgroup (n=22)	Exogenous subgroup (n=14)	p-value
Age (years)	33.2 ±6.4	38.2±7.6	0.234
BMI (kg/m^2^)	24.3 ± 1.5	24.8 ± 1.6	0.609
Duration of disease (months)	12 ± 6.9	6.2 ± 5.4	0.004
TSH (mIU/ml)	0.26±0.07	0.29±0.09	0.149
fT4 (ng/dl)	1.3± 0.18	1.4±0.15	0.128
Corrected Calcium (mg/dl)	9.13 ± 0.26	9.12 ± 0.26	0.864
Phosphorous(mg/dl)	2.8±0.35	3.0±0.28	0.844
25 hydroxy vitamin D (ng/ml)	31.8 ±3.8	27.4± 4.8	0.721

**Table 2:** Bone mineral density and C telopeptide of the two groups The data has been represented as Mean±SD, etc. The p-value is considered significant at <0.05. BMD: Bone mineral density

	Endogenous (n=22)	Exogenous (n=14)	p-value
BMD spine (g/cm^2^)	0.9 ±0.07	0.92±0.09	0.445
BMD hip (g/cm^2^)	0.79 ± 0.19	0.83± 0.17	0.142
C Telopeptide (pg/ml)	380 ± 175.6	223.17 ± 143.6	0.008

Table 3 illustrates that there is no significant co-relation observed between TSH and lumbar 1-4 Z score, femur neck Z score, BMD femur, BMD lumbar 1-4, and C telopeptide.

**Table 3:** Co-relation between thyroid-stimulating hormone (TSH) with lumbar 1-4 Z score, femur neck Z score, bone mineral density (BMD) femur, BMD lumbar 1-4, and C telopeptide The p-value is considered significant at <0.05.

Co-relation of TSH	
Lumbar Z score	r=-0.12 p=0.46
Femur neck Z score	r=-0.24 p=0.72
BMD femur	r=-0.37 p=0.45
BMD Lumbar	r=-0.19 p=0.15
C Telopeptide	r=-0.17 p= 0.74

Table 4 reveals no significant co-relation between thyroid stimulating hormone (free T4) and lumbar 1-4 Z score, femur neck Z score, BMD femur, and BMD lumbar 1-4.

**Table 4:** Co-relation between thyroid stimulating hormone (free T4) with lumbar Z score, femur neck Z score, bone mineral density (BMD) femur, and BMD lumbar vertebrae The p-value is considered significant at <0.05.

Co-relation of fT4	
Lumbar 1-4 Z score	r=-0.12 p=0.65
Femur neck Z score	r=-0.21 p=0.68
BMD femur g/cm2	r=-0.43 p=0.28
BMD L1-4 g/cm2	r=-0.19 p=0.47
C-Telopeptide	r= -0.58 p=0.52

## Discussion

Despite the ongoing discussion regarding the influence of SCH on bone, our cross-sectional study found that the BMD in premenopausal women with both endogenous and exogenous SCH was not significantly reduced compared to the Z- score of the reference population in the short term.

In a study by Saler et al. on 86 premenopausal women with SCH, there was no significant reduction in BMD in either exogenous or endogenous subgroups. The Z-score (femur and L1-4) of the study group was -0.15 ± 1.15 and -0.23 ± 1.03, respectively. The Z-score of the control group was -0.39 ± 1.08 and -0.55 ± 0.98, respectively. The differences between the groups were not statistically significant (p=0.14, 0.34, respectively) [11].

Kumeda et al. studied 19 women with SCH with both exogenous and endogenous etiology. Serum bone alkaline phosphatase (B-ALP), bone formation markers, and urinary excretions of pyridinoline (U-PYD) and deoxypyridinoline (U-DPD), which are bone resorption markers, were significantly higher in the TSH-suppression group than in the TSH-normal group (B-ALP, p< 0.05; U-PYD, p< 0.001; U-DPD, p< 0.001) [12]. 

A study by Gürlek et al. on 15 women with endogenous SCH whose persistence was confirmed three months apart reported no significant reduction in BMD in the short term [10]. De Rosa et al. demonstrated that the administration of levothyroxine significantly elevated bone mineral turnover, potentially leading to a reduction in BMD in both pre- and post-menopausal women [13]. It is widely acknowledged that premenopausal women typically experience little to no loss of BMD [14]. The primary factor accepted for this minimal loss or absence of bone mass reduction in premenopausal women is considered to be sufficient estrogen production [15].

However, most of the studies suggesting a decrease in BMD in women with SCH showed the data of either postmenopausal women or women with suppressed TSH values under L-T4 treatment [16]. The current trend of increasing vitamin D supplementation in the subcontinent may have a protective role in these patients [17].

The telopeptides are cleaved during osteoclastic resorption of bone, resulting in their liberation into the circulation at a rate proportional to bone resorption activity. CTx is the specific product of cathepsin K-mediated bone resorption, as direct digestion of bone with cathepsin K. CTx is small enough to be renally cleared, and accordingly, serum/plasma and urine are both suitable sample types. In clinical practice, CTx is most commonly analyzed in serum [9].

In our study, the mean serum CTx was 380 ± 175 and 223 ± 143 pg/ml in endogenous and exogenous subgroups respectively and the difference was significant. Although there was a significant difference in C terminal telopeptide between the endogenous and exogenous subgroups, it may be attributed to a longer duration of SCH in the endogenous subgroup.

In a study on BTMs in normal premenopausal south Indian women by Sahana et al., the 2.5th to 97.5th centile was reported between 363 and 457 pg/ml. In our study, the mean serum CTx was 380 ± 175 and 223 ± 143 pg/ml in endogenous and exogenous subgroups respectively and the difference was significant. The significance of difference and normative data for our population needs to be confirmed in further well-matched prospective cohorts [18].

One limitation of our study was the small sample size, although very few studies are available from India in this area. The duration of the SCH state ascertained by history may not be accurate, especially in the exogenous subgroup. There was no control group. Future studies with confirmation of persistent subclinical thyrotoxic state with a longer duration of follow-up are needed. Qualitative bone imaging methods like trabecular bone score may provide additional detailing in this subgroup of patients.

## Conclusions

Our cross-sectional study of premenopausal women with SCH, irrespective of the underlying cause (endogenous or exogenous), did not reveal any significant reduction in BMD at the hip or spine. In the short term, a subclinical thyrotoxic state may not have a harmful effect on bone health in young females with sufficient levels of serum 25-hydroxy-vitamin D. Further investigations with the verification of a sustained subclinical thyrotoxic condition through an extended follow-up period are essential.
